# Adenosine cardiac magnetic resonance: follow-up of patients with very high cardiovascular risk

**DOI:** 10.1186/1532-429X-17-S1-P193

**Published:** 2015-02-03

**Authors:** Alberto Esteban-Fernández, Isabel Coma-Canella, Gorka Bastarrika-Aleman, Joaquín Barba-Cosials, Nahikari Salterain-Gonzalez, Pedro M Azcárte-Aguero

**Affiliations:** Cardiology Department, Clínica Universidad de Navarra, Pamplona, Spain; Radiology Department, Clínica Universidad de Navarra, Pamplona, Spain

## Background

Stress cardiac magnetic resonance with adenosine (CMR-A) is a valid test to rule out myocardial ischaemia. We follow-up a cohort of patients with CMR-A due to suspected myocardial ischaemia, considering patients with very high cardiovascular risk.

## Methods

We included all patients with CMR-A between June 2009 and November 2012, considering two groups: those with a very high cardiovascular risk (prior myocardial infarction or/and Diabetes mellitus) and the rest of the patients. The follow-up was done in outpatient cardiology clinic or by phone. We analyse free-event survival considering: acute coronary syndrome (ACS), death for any cause, admission for heart failure (HF) or necessity of revascularization as endpoints. The statistical analysis was made with SPSS 20.0.

## Results

The follow-up of 239 patients (180 male) was done. 134 (56%) were re-classified as high cardiovascular risk patients and 105 (44%) as non-high cardiovascular risk ones. The basal characteristics of each group are summarize in table 1.

**Table 1:** Characteristics of the patients with CMR-A to rule out myocardial ischaemia considering cardiovascular risk

	High cardiovascular risk (n=134)	Non-high cardiovascular risk (n=105)
Age-years old	68.0±8.3	63.8±11.8
Diabetes mellitus-no (%) Basal glucose (mg/dL) HbA1c (%)	93 (69.4) 125±41 7.0±1.4	- 103±33 6.1±1.3
No tobacco history-no (%)	48 (35.8)	55 (52.4)
Arterial hypertension-no (%)	97 (72.4)	69 (65.7)
Dyslipidaemia-no (%)	104 (77.6)	53 (50.5)
BMI (kg/m2)	29.8±6.1	27.1±4.7
Previous ictus-no (%)	12 (9.0)	4 (3.1)
Peripheral arterial disease-no (%)	33 (24.6)	7 (6.7)
Previous myocardial infarction-no (%) PCI revascularization-no (%) CABG revascularization-no (%)	68 (50.7) 51 (38.1) 29 (21.6)	
Result in CMR-A Positive-no (%) Negative-no (%)	- 56 (41.8) 78 (58.2)	- 27 (25.7) 78 (74.3)
Events in follow-up-no (%) ACS-no (%) Death for any cause-no (%) Admission for HF-no (%) Revascularization-no (%)	51 (38.1) 21 (15.7) 8 (6) 2 (1.5) 20 (14.9)	17 (16%) 5 (4.8) 8 (7.6) 3 (2.9) 1 (1)

CMR-A was positive for myocardial ischaemia in 83 patients (35%) and negative in 156 (65%). The follow-up median was 25 months, with events in 68 patients. The results of the test and the events during the follow-up in each group are attached in table 1.

The analysis of Kaplan-Meier survival curves (1 and 2), considering the cardiovascular risk and the result of the test, showed statistical differences only in very high cardiovascular risk patients (Long Rank test; p=0.024).

**Figure 1:**
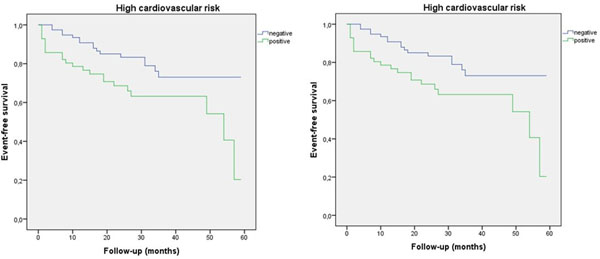
Figure 1

## Conclusions

In this cohort of patients with very high cardiovascular risk, those with a negative result have fewer events in the follow-up. CMR-A allows a better classification of the global cardiovascular risk

## Funding

There is not any funding to support this trial.

